# The power of competition: Effects of social motivation on attention, sustained physical effort, and learning

**DOI:** 10.3389/fpsyg.2015.01282

**Published:** 2015-09-01

**Authors:** Brynne C. DiMenichi, Elizabeth Tricomi

**Affiliations:** Department of Psychology, Rutgers University, Newark, NJ, USA

**Keywords:** competition, social motivation, learning, memory, attention, sustained effort

## Abstract

Competition has often been implicated as a means to improve effort-based learning and attention. Two experiments examined the effects of competition on effort and memory. In Experiment 1, participants completed a physical effort task in which they were rewarded for winning an overall percentage, or for winning a competition they believed was against another player. In Experiment 2, participants completed a memory task in which they were rewarded for remembering an overall percentage of shapes, or more shapes than a “competitor.” We found that, in the physical effort task, participants demonstrated faster reaction times (RTs)—a previous indicator of increased attention—in the competitive environment. Moreover, individual differences predicted the salience of competition’s effect. Furthermore, male participants showed faster RTs and greater sustained effort as a result of a competitive environment, suggesting that males may be more affected by competition in physical effort tasks. However, in Experiment 2, participants remembered fewer shapes when competing, and later recalled less of these shapes during a post-test, suggesting that competition was harmful in our memory task. The different results from these two experiments suggest that competition can improve attention in a physical effort task, yet caution the use of competition in memory tasks.

## Introduction

Social motivation has been defined as a drive for a particular goal based on a social influence (Hogg and Abrams, 1990). Although research has examined correlative relationships between competition and learning (Dweck and Leggett, 1988; Zimmerman, 1989; Oldfather and Dahl, 1994; Wentzel, 1999), few studies have examined how the presence of a competitor directly influences motivation, effort, and memory. In Burguillo (2010) found that implementing competition-based games in a classroom improved course performance. One might therefore assume that competition may directly improve some aspect of the memory process; yet, it is unclear whether competition directly affects attention, effort, or memory.

Recent research has shown that the presence of a competitor can increase physical effort over both short (Le Bouc and Pessiglione, 2013) and long durations (Kilduff, 2014). Competitiveness has also been shown to increase physical motivation, such as motivation to practice a sport (Frederick-Recascino and Schuster-Smith, 2003). A better understanding of how competition improves performance may help shed light on how to improve cognitive performance (e.g., memory in the classroom). For example, if the presence of a competitor affected attention, we may expect to see an effect at encoding, since attention is one of many necessary components for accurate encoding (Craik et al., 1996; Anderson et al., 2000; Fernandes and Moscovitch, 2000). However, if the presence of a competitor is affecting memory retention, we may expect a difference regarding long-term memory, but not short-term memory. Furthermore, competition could affect components of memory without affecting attention at all.

There may also be individual differences in the magnitude and direction of competition’s effect on performance. Individual differences exist in a variety of domains, especially those involving motivation (Duckworth et al., 2007; Maddi et al., 2012). For example, previous research has found that individual differences in normative goals—i.e., wanting to perform better than others (Grant and Dweck, 2003)—have been shown to predict performance on ostensibly difficult tasks (Swanson and Tricomi, 2014), suggesting that individual differences may be at play when examining competition’s effect on effort, attention, and memory. Also, competition may affect elements of effort and elements of memory in different ways. For example, if competition does indeed have an effect on attention, competition could have a varying effect depending on attentional load. In accordance with the Yerkes and Dodson (1908) law, one might expect that competition may improve performance in situations requiring a low attention load, but not in learning environments requiring high attentional load.

Additionally, research has yet to examine the potential social stigma associated with competition, or in other words, whether being competitive is viewed as a negative personality trait. Moreover, previous research regarding illusory superiority has found that individuals tend to rate themselves as having significantly more positive personality traits than the rest of the population, including traits such as trustworthiness, honesty, good-humor, and patience (Hoorens, 1995). Furthermore, previous research has found that the majority of individuals rate themselves as significantly less likely to act selfishly than the rest of the general population (Pronin et al., 2002), as well as drive better (Horswill et al., 2004) than the rest of the general population. Since individuals tend to have unrealistically positive reflections of themselves, participants may tend to rate themselves as having less competitive behaviors—if competitive behavior is viewed as a socially negative trait—in order to continue to view themselves in a positively-skewed light.

Experiment 1 examined the effect of social motivation on a physical effort task. Experiment 2 examined the effect that the presence of a competitor can have on working memory and long-term memory. We hoped to gain insight regarding competition’s effect on effort, attention, and memory, as well as individual differences in competitive performance and the likely possibility of a social desirability bias regarding competitive habits.

## Experiment 1

Experiment 1 examined whether competition affects physical effort. Specifically, we wondered if competition would affect sustained effort on an isolated, simple physical task, or if competition affects some other mechanism necessary for successful performance regarding physical effort, such as attentional control. Le Bouc and Pessiglione (2013) found that, when participants believed they were competing, they increased physical effort, suggesting that social factors often increase motivation. However, research has yet to parse the mechanisms at play in social motivation and physical effort. For example, does competition increase effort at the attentional level, or does the presence of a competitor increase sustained effort over time? Previous research has suggested that reaction times (RTs) are indicative of an individual’s level of selective attention (Eason et al., 1969; Stuss et al., 1989; Prinzmetal et al., 2005), while sustained press rates have been regularly implicated as a means for measuring sustained effort over time (Maatsch et al., 1954; Treadway et al., 2009). We also wanted to examine the possibility of individual differences in physical effort in the presence of a competitor, and the possibility of gender differences in the saliency of social motivation.

### Method

#### Participants

One hundred and twenty-nine undergraduates from Rutgers University’s Newark campus participated in the study, which was approved by the Rutgers IRB. Participants received course credit for their participation, and were told upon arriving they would be eligible to earn $1–3 in bonus money in addition to course credit. Participants entered the lab and were introduced to a fellow “participant” they would later be interacting with—a same or opposite sex confederate. After obtaining written informed consent from the participant, the experimenter brought the confederate into a testing room and waited for about 5 min, the expected time for the confederate to complete the practice session of the task. Participants then completed a practice version of the task, the actual task, and a battery of surveys, including demographic information. After completing the surveys, participants were probed about whether or not they believed they were actually competing against another individual and if they believed the confederate was a real participant. Then, participants were debriefed about the confederate and real purpose of the task. Seven participants were removed for not believing the manipulation, and two participants were removed for failing to complete the task in its entirety. Analyses were thus performed on the remaining 120 participants.

#### Effort Bar Task

Participants completed an effort bar task in the form of a computerized carnival water gun game. Participants saw a fixation cross with a 3–7 s jitter, then were required to press the “x” key to move the effort bar (in this case, in the form of a “water tube”). If participants pressed the “x” key before the water tube appeared, the jitter reset. Participants were required to press between a randomly generated requirement of 5 and 30 times to fill the effort bar in order to win the trial. Participants had to press at an average rate of 150 ms to fill the tube with water in time to win the round, with an extra 350 ms to account for the expected first press time. This time amount was decided due to the results of a pilot study that found that participants had an average first press of 350 ms and press rate (excluding the first press) of one press per 150 ms. Titrating the task at this rate led to the expectation that participants would win an average of 50% of trials. We analyzed participants’ first press RTs as a measure of their attention to the task (Eason et al., 1969; Stuss et al., 1989; Prinzmetal et al., 2005), as well as their sustained press rate over the span of the task, which provided us a measure of sustained effort (Maatsch et al., 1954; Treadway et al., 2009).

***“Self” condition***

In the “self” condition, participants were told they were playing against the clock, and that if they could win 2/3 of the games (trials) played in this round, they would be granted $1 in addition to their course credit. There were 100 trials per condition (200 trials total). Participants were given immediate feedback after each trial as to whether they won, and were immediately told at the end of each self and each competition condition if they won the bonus money. Conditions were counterbalanced across participants to prevent order effects.

***“Competition” condition***

**FIGURE 1:**
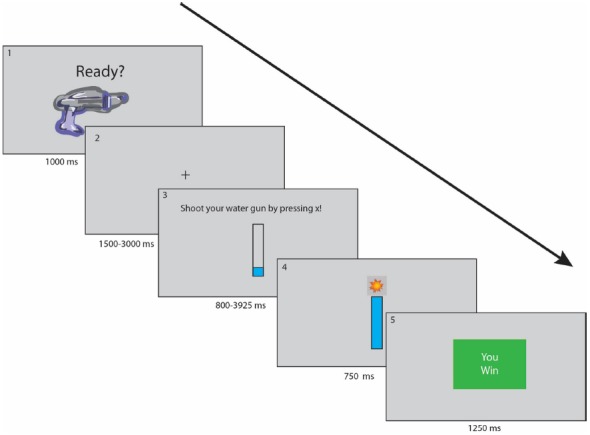
**Experiment 1 task depiction.** Participants saw a preparation screen (Slide 1) for 2 s, then a fixation jittered for 1.5–3 s (Slide 2). Participants pressed the x key repeatedly when they saw the effort bar appear; time was varied by the number of required presses (Slide 3). Participants were told if they filled the effort bar in time (Slide 4) and were given feedback regarding their performance (Slide 5).

In the competition condition, participants were told they were playing against the other “participant” they met earlier (again, a confederate), and would be granted an additional $1 if they could beat their competitor in more of the games. At the end of each game, they were told whether they or the other player won the game, and were told who won the bonus at the end of each self and each competition condition. If participants won 2/3 of the games in a particular condition, they were granted the bonus. Each participant completed both conditions, and conditions were counterbalanced across participants to account for possible order effects. Task depiction is illustrated in Figure 1.

#### Surveys

We administered several surveys to investigate potential individual differences and their relationship to task performance.

***Hypercompetitive Attitude Scale (HAS)***

The HAS examines individual differences in general hypercompetitive attitude (Ryckman et al., 1990). The HAS asks participants to reflect on habits and traits that may be associated with a competitive personality (e.g., “I can’t stand to lose an argument.”).

***Personal Development Competitive Attitude Scale (PDCAS)***

The PDCAS examines if individuals regard competition as a means of improving personal development (Ryckman et al., 1996) The PDCAS reflects on preference for situations in which competition may improve their performance (e.g., “I enjoy competition because it gives me a chance to discover my abilities.”).

***Marlow-Crowne Social Desirability Scale (SDS)***

We included the SDS (Crowne and Marlowe, 1960) to measure possible bias in responding, whether it be because participants have unrealistic representations of their own traits, or because of a desire to please the experimenter. This questionnaire examines the extent to which a subject may positively skew their survey responses to represent themselves in a positive manner, and requires a “true or false” response to items such as “I am always courteous, even to people who are disagreeable.” The SDS has been previously used to detect the tendency of participants to have unrealistically positive representations of their own traits (Zerbe and Paulhus, 1987; Paulhus, 1991; DiMenichi and Richmond, 2015). Because Ryckman et al. (1990) found that HAS was also correlated with high aggression, we were unsure whether participants would be likely to admit the extent of their competitive natures. Furthermore, research has yet to examine whether or not individuals view competition as a negative personality trait, and a correlation with the HAS and SDS would suggest this.

#### Analyses

***Main analyses***

A within-subjects *t*-test examined differences between the first-press RTs in the self condition and the first-press RTs in competition condition. A within-subjects *t*-test also examined differences between the sustained press-rates in the self condition and the sustained press-rates in the competition condition.

***Individual differences analyses***

Pearson correlations examined the relationship between trait competitive tendencies (HAS and PDCAS), first-press RTs, and sustained press-rates from the competition condition and the self condition. Pearson correlations also examined relationships between survey scores and scores on the SDS in order to examine possible biases in participants’ responding, as well as if competitive habits are viewed as a socially-negative trait. We used a Bonferroni corrected significance threshold of *p* = 0.017 (0.05/3 scales) and interpreted correlations with *p*-values between 0.018 and 0.05 with caution.

***Gender differences analyses***

Between-subjects *t*-tests examined gender differences in performance and on the survey measures (HAS, PDCAS, and SDS) used in our experiment. Two-way analyses of variance (ANOVAs) also examined the effects of the factors gender and confederate gender on competitive first-press RT (first-press RT in the competition condition minus the first-press RT in the self condition) and competitive press rate (press rate in the competition condition minus the press rate in the self condition). Within-subject *t*-tests for each group individually also examined differences in performance across conditions (30 participants per group).

### Results and Discussion

**FIGURE 2:**
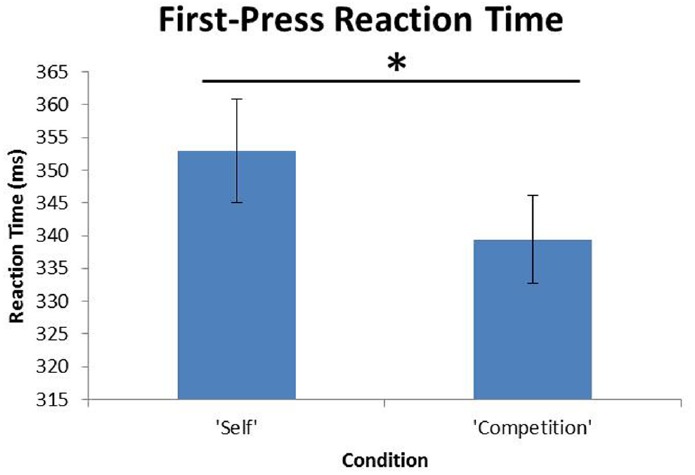
**Results from Experiment 1.** Participants’ first press reaction times (RTs) were significantly faster in the competition condition than the self condition. Error bars reflect standard errors of the means. *Significant at *p* < 0 .05.

A paired-samples *t*-test revealed that participants’ first presses—i.e., immediate RTs on the task—were significantly faster in the competition condition (*M* = 339.43 ms, SD = 72.96) than in the self condition [*M* = 352.89, SD = 86.84; *t*(119) = –2.62, *p* = 0.010, Cohen’s *d* = 0.24], suggesting that participants demonstrated greater attentional focus on the task when they believed they were competing against another participant (Figure 2). There were no other significant findings regarding press rate, score, and condition, suggesting that competition affected attentional focus on the task, but not sustained physical effort over time.

Scores on the SDS were significantly negatively correlated with scores on the HAS (*r* = –0.367, *p* < 0.001), suggesting that overt competition may be implicitly viewed as a negative personal quality by most individuals. There was no significant relationship between scores on the SDS and scores on the PDCAS, suggesting that the PDCAS may be immune to participants’ tendencies to paint themselves in a positively-skewed manner. Scores on the PDCAS were significantly correlated with faster RTs of the first press in competition condition (*r* = –0.239, *p* = 0.008), suggesting that individuals who view competition as a means for personal development may have greater attentional focus in the presence of a competitor. However, there was no significant relationship between scores on the PDCAS and first press RT in the self condition, which is consistent with the idea that competitive personality traits should not affect performance in an environment with no competition.

**FIGURE 3:**
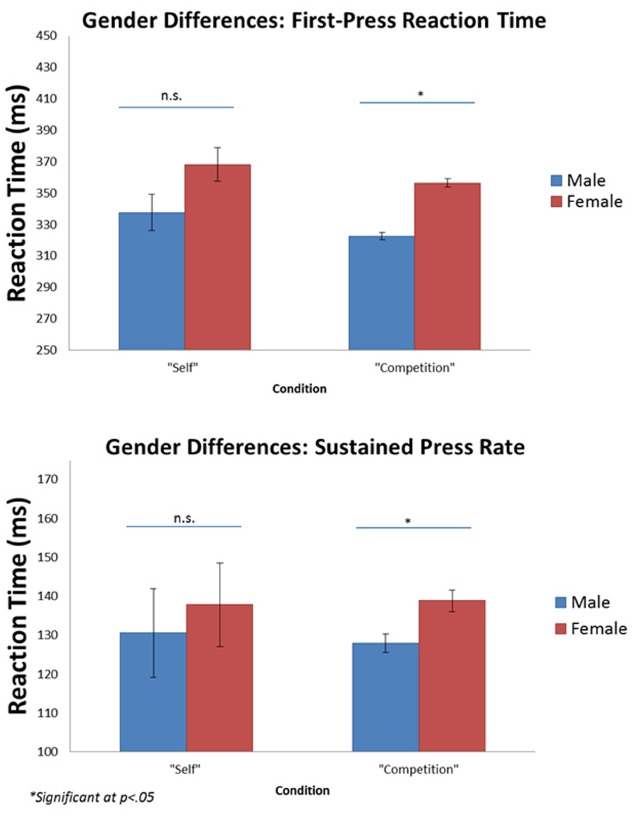
**Gender differences from Experiment 1.** Males had significantly faster first press reaction times and significantly faster press rates in the competition condition compared to female’s first press reaction times and press rates in the competition condition. However, there was no significant gender difference in the self condition. Error bars reflect standard errors of the means.

Men also scored significantly higher on the PDCAS (*M* = 51.59, SD = 9.65) than women [*M* = 46.62, SD = 11.68; *t*(118) = 2.53, *p* = 0.012, Cohen’s *d* = 0.46], suggesting that men may view competition as a greater motivation for improving skills pertaining to personal development. Additionally, male participants demonstrated significantly faster first press RTs in the competition condition than female participants’ first press RTs in the competition condition [male *M* = 323.23, SD = 71.44; female *M* = 335.09, SD = 71.53; *t*(118) = –2.44, *p* = 0.016, Cohen’s *d* = 0.17] Furthermore, male participants also had faster sustained press rates in the competition condition (*M* = 128.36, SD = 16.01) when compared to females participants’ press rates in the competition condition [*M* = 138.26, SD = 11.98; *t*(118) = –3.84, *p* < 0.001, Cohen’s *d* = 0.70]. However, there were no significant gender differences involving first press RT in the self condition or press rate in the self condition. Furthermore, when examining male participants’ sustained press rate performance, there was no significant difference between press rate in the competition and self conditions. See Figure 3 for gender difference results across conditions. A two-way ANOVA with the factors participant gender and confederate gender did not reveal a significant main effect of confederate gender [*F*(3) = 0.48, *p* = 0.695] or interaction of gender by confederate gender [*F*(42) = 0.63, *p* = 0.825 Cohen’s *d* = 0.08] on competitive first-press RTs. Also, a two-way ANOVA with the factors participant gender and confederate gender did not reveal a significant main effect of confederate gender [*F*(3) = 0.75, *p* = 0.528] or interaction of gender by confederate gender [*F*(42) = 1.25, *p* = 0.209, Cohen’s *d* = 0.10] on competitive press rate. Overall, these findings suggest that men were significantly more socially motivated in the presence of another competitor, at least in terms of attention in a physical effort task.

Our findings from Experiment 1 suggest that competition had an effect on participants’ attention to our task. We did not find a significant relationship between competition and sustained physical effort in our task, suggesting that competition may have a more cloudy relationship with physical effort than our task was able to provide. Furthermore, our results suggest that there are predictable individual differences in competition’s influence on attention, although reflection on these individual differences may be vulnerable to a bias of individuals to paint themselves in an overly positive light, whether implicitly or explicitly (e.g., due to task-demand characteristics or the presence of an experimenter). Also, our findings show that men’s attention on a physical effort task may be more influenced by the presence of a competitor than women’s.

## Experiment 2

Because Experiment 1 found that competition increased attention, Experiment 2 examined whether the presence of a competitor enhanced working memory as well as memory retention, mechanisms that both rely heavily on attention. Specifically, we examined whether competition would inspire greater performance on a memory task and, if so, what mechanisms are responsible.

### Method

#### Participants

One hundred and twenty-four undergraduates from Rutgers University’s Newark campus participated in the study, which was approved by the Rutgers IRB. Participants received course credit for their participation, and were told upon arriving they would be eligible to earn $1–3 in bonus money in addition to course credit. Experiment 2 followed the same laboratory format as Experiment 1: upon entering the lab, participants were introduced to another “participant” they would later be interacting with—a same or opposite sex confederate. After obtaining written informed consent from the participant, the experimenter brought the confederate into a testing room and waited for about 5 min, the expected time for the confederate to complete the practice session of the task. Participants then completed a practice version of the task, the actual task, a surprise recall task, and a battery of surveys, including demographic information. After completing the surveys, participants were probed for task believability and debriefed about the confederate and real purpose of the task. Four participants were removed from the sample for not believing that the confederate was a participant. Analyses were performed on the remaining 120 participants (60 females).

#### Working Memory Task

**FIGURE 4:**
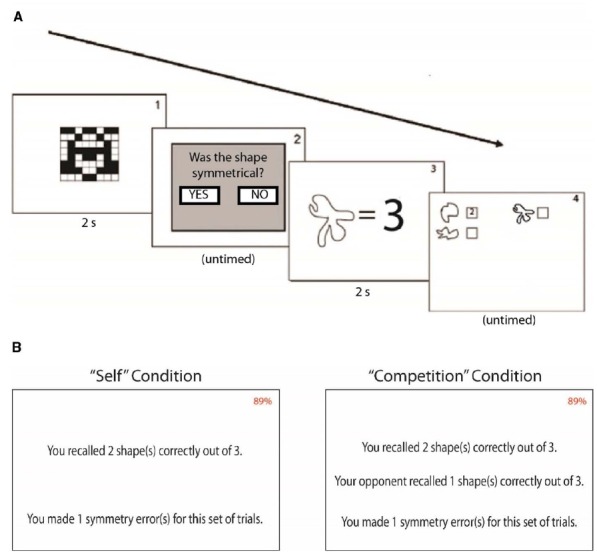
**Experiment 2 task depiction. (A)** Participants were shown a matrix for 2 s (Slide 1) and asked to decide if the shape was symmetrical (Slide 2). Participants were then shown a novel shape paired with a number (1, 2, or 3) for 2 s, and were asked to memorize this association (Slide 3). After three rounds (of Slides 1–3), participants were asked to recall the numbers associated with the shapes. **(B)** Subjects were given immediate feedback for 6 s regarding their performance on the previous round. In the self condition (left), subjects were informed about how many shapes they recalled correctly. After a 2 s delay, they also saw the number of symmetry errors they made on this trial, and the total percentage of symmetry problems answered correctly throughout the condition (top right corner—subjects were required to answer at least 85% of symmetry problems correctly in order to receive the monetary bonus). In the competition condition (right), subjects were also given feedback about the number of shapes their “opponent” remembered correctly—a randomly generated number from 0 to 3. After a 2 s delay, they were also given feedback about their symmetry performance.

Our working memory task was adapted from (Redick et al., 2012). Participants decided if a matrix was symmetrical or not, and then were presented with a line drawing of an abnormal shape, along with a number (1 through 3). See Figure 4 for task depiction. They were asked to memorize the association between the shape and the number. Novel shapes were taken from Endo et al.’s (2001) Novel Shape database. After three different matrices and shapes were shown, participants were shown a recall screen with the shapes from the trial, and asked to recall the numbers associated with the shapes they were just shown. Each condition contained 12 rounds with 18 novel shapes randomly assigned to each condition, and each round was shown twice because of a later recall task. Each participant completed both conditions, and shapes in the “self” condition were not repeated in the “competition” condition (and *vice versa*). Conditions were counterbalanced across participants to prevent order effects, and shapes in each condition were counterbalanced across participants, in case shapes in one condition were somehow more difficult than shapes in another condition.

***“Self” condition***

In the self condition, participants were given feedback about their performance directly after the recall screen: they were told how many shapes they recalled correctly out of three, as well as how many symmetry problems they answered correctly. They were also given the running total percentage of correct symmetry problems for the entire condition. Participants viewed feedback for 6 s after each round, and were told that if they could remember a total average of 2/3 shapes across all rounds for this condition, they would be given a $1 bonus in addition to their course credit. They were also told that in order to receive the bonus, they were required to complete the task with a symmetry matrix accuracy of at least 85%. Inclusion of the symmetry task also allowed us to examine if effort on the task varied across conditions, since this section of the task did not have a memory component.

***“Competition” condition***

In the competition condition, after each recall screen, participants were given feedback about how many shapes they correctly recalled out of three, as well as feedback about their “competitor’s” performance. Competitor performance was randomly generated out of 3, and averaged out to be 2/3 across the entire condition, making the task goal equivalent across both the self and competition conditions. After a 2 s delay, participants were also given feedback about symmetry matrices errors for the round. This delay was issued in order to present the same amount of information across conditions, therefore making cognitive load on working memory more equal across conditions. Total recall viewing time was 6 s after each round. Participants were told if they could recall more associations than the other participant on the most rounds—as well have a symmetry matrix accuracy of at least 85%—they would get a $1 bonus at the end of the condition. Condition feedback is depicted in Figure 1B.

***Recall task***

In a surprise recall task that followed the working memory task, participants were again asked to recall each number associated with each shape. Shape order was randomized to prevent order effects.

#### Analyses

***Main analyses***

A within-subjects *t*-test examined differences between the number of shapes remembered in the self condition and the number of shapes remembered in competition condition of the working memory task. A within-subjects *t*-test also examined whether there were differences in subsequent memory between the two conditions, i.e., whether there were differences between the number of shapes originally learned in the self condition and the number of shapes originally learned in the competition condition that were correctly recalled on the surprise recall posttest. To compare any differences in immediate attention across conditions, a within-subjects *t*-test examined RT to the first symmetry problem between the two conditions. We also subtracted each participant’s total number of shapes remembered during the self condition of the working memory task from their total number of shapes remembered during the competition condition of the working memory task, and deemed this score each participant’s “competitive performance score.” A positive number would indicate better performance on the competition condition of our task. We also repeated the process for post-test scores. A linear regression examined if competitive performance scores predicted competitive recall scores, in order to examine if recall scores on the post-test were the result of learning during the working memory task. If there was no significant relationship between competitive performance scores and competitive recall scores, we would assume that competition increased effort on our task, but not immediate long-term memory. Self scores were subtracted from competition scores in order to account for general memory ability on the task.

***Individual differences analyses***

Pearson correlations (Bonferroni corrected for multiple comparisons, α = 0.017) examined the relationship between trait competitive tendencies (HAS and PDCAS) and working memory scores from the competition condition and self condition, as well as recall scores. Pearson correlations also examined relationships between survey scores and scores on the SDS in order to examine possible biases in participants’ responding, as well as if competitive habits are viewed as a socially-negative trait. A partial Pearson correlation also examined relationships between trait competitive tendencies and performance while controlling for scores on the SDS.

***Gender differences analyses***

Between-subjects *t*-tests examined gender differences in performance, recall, and on the survey measures (HAS, PDCAS, and SDS) used in our experiment. Two-way ANOVAs also examined the effect of the factors gender and confederate gender on competitive performance and competitive recall scores. Furthermore, within-subject *t*-tests for each group individually examined differences in performance across conditions (30 participants per group). Partial Pearson correlations controlling for SDS also examined the relationship between trait competitive tendencies (HAS and PDCAS) and working memory scores from the competition condition, self condition, and recall conditions in order to examine if the presence of a same- or opposite-sex confederate is salient enough to override state tendencies.

### Results and Discussion

A paired-samples *t*-test revealed that participants performed significantly better in the self condition (*M* = 28.78, SD = 6.87) than the competition condition [*M* = 26.72, SD = 6.24; *t*(119) = 3.85, *p* < 0.001, Cohen’s *d* = 0.31] during the working memory task. There was no significant difference between symmetry error rates across conditions, as well as no significant difference in RT to the first symmetry problem across conditions, suggesting that competition did not affect participants’ expended effort on the task, but specifically affected working memory performance. Furthermore, a paired-samples *t*-test revealed that participants later recalled more shapes on the post-test learned in the self condition (*M* = 10.61, SD = 4.40) than in the competition condition [*M* = 8.76, SD = 3.34; *t*(119) = 4.06, *p* < 0.001, Cohen’s *d* = 0.37]. A linear regression revealed that competitive performance scores significantly predicted competitive recall scores [β = 0.25, *t*(119) = 3.34, *p* = 0.005], and competitive performance scores also explained a significant proportion of variance in competitive recall post-test scores [*R*^2^ = 0.09, *F*(1,118) = 11.15, *p* = 0.001], suggesting that recall scores on the post-test were the result of learning during the working memory task. If there was not a significant relationship between competitive performance scores and competitive recall scores, we would assume that competition increased effort on our task, but not immediate long-term memory.

A Pearson correlation on our survey data revealed a marginally significantly positive association between scores on the PDCAS and performance in the competition condition (*r* = 0.17, *p* = 0.061), but not in the self condition. Because scores on the SDS were again relatively high in our sample—participants answered an average of 55.25% of questions in a “socially desirable” manner—we conducted a partial correlation that revealed that, when controlling for SDS, PDCAS scores were marginally significantly associated with performance during the competition condition (*r* = 0.18, *p* = 0.048). However, after adjusting for multiple comparisons, this finding was no longer significant.

As predicted, SDS scores were again significantly negatively correlated with scores on the HAS (*r* = –0.367, *p* < 0.001), replicating our findings from Experiment 1 and again suggesting that our participants’ self-reflections of their own competitive habits may be skewed. Since HAS contains questions pertaining to direct competitive tendencies, overt competitiveness may be considered a negative personality trait by most individuals. Furthermore, although HAS scores were significantly associated with PDCAS scores (*r* = 0.304, *p* < 0.001), PDCAS scores were not significantly associated with SDS scores, again suggesting that competition as a means for personal development may be viewed more positively than overt competitive behavior and beliefs.

**FIGURE 5:**
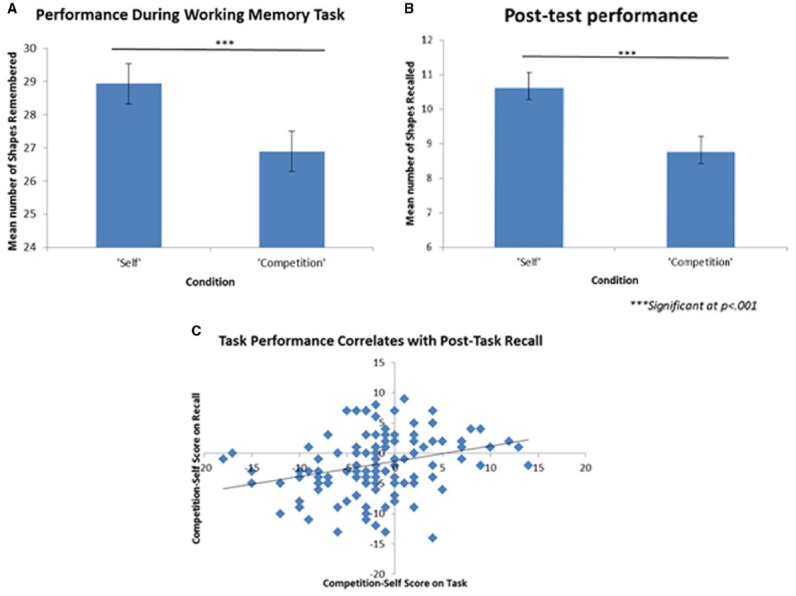
**Results of Experiment 2. (A)** Participants remembered significantly more shapes during the task in the “self” condition than the “competition” condition. **(B)** Participants later recalled more shapes learned in the “self” condition than the “competition” condition. **(C)** “Competitive performance scores” (score on “self” condition subtracted from score on “competition” condition) significantly predicted “competitive recall scores” (shapes from the “self” condition successfully recalled on the post-test subtracted from shapes from the “competition” condition successfully recalled), suggesting that our working memory task produced significant immediate long-term learning. In this graph, a positive score signifies more competitive score. Error bars reflect standard errors of the means.

Although the men in our sample again scored significantly higher on the PDCAS (*M* = 56.03, SD = 13.26) than women [*M* = 49.27, SD = 14.76; *t*(118) = 2.87, *p* = 0.005, Cohen’s *d* = 0.48], there were no significant differences regarding gender and task performance or recall. We also examined the results with respect to the gender of the confederates. A two-way ANOVA with the factors participant gender and confederate gender did not reveal a significant main effect of confederate gender [*F*(3) = 1.48, *p* = 0.229] or an interaction of gender by confederate gender [*F*(42) = 1.09, *p* = 0.735, Cohen’s *d* = 0.36] on competitive performance scores, nor did a two-way ANOVA with the factors participant gender and confederate gender reveal a significant main effect of confederate gender [*F*(3) = 2.28, *p* = 0.088] or an interaction of gender by confederate gender [*F*(42) = 1.73, *p* = 0.066, Cohen’s *d* = 0.45] on competitive recall scores. Furthermore, pair-wise *t*-tests revealed that neither men nor women who competed against male confederates showed any significant difference in self vs. competitive performance. Yet, male participants who competed against female confederates performed significantly worse [*t*(29) = 3.54, *p* = 0.001, Cohen’s *d* = 0.65] and female participants who competed against female confederates performed marginally significantly worse [females: *t*(29) = 1.91, *p* = 0.066, Cohen’s *d* = 0.35] while they believed they were competing than when they were not competing. Furthermore, both male and females participants who competed against female confederates later recalled significantly fewer shapes learned in the competition condition [males: *t*(29) = 3.38, *p* = 0.002, Cohen’s *d* = 0.62; females: *t*(29) = 3.00, *p* = 0.006, Cohen’s *d* = 0.55]. All groups contained equal *n*’s of 30 participants in each group. Although one could suggest that a significant difference among participants who believed they were competing against females may have resulted because these participants were exerting less effort against female competitors, there were no significant group differences regarding symmetry errors, suggesting that effort on the task was equal across groups, while memory on the task was hindered in those participants who faced female competitors. Details regarding group differences are depicted in Figure 5.

When controlling for social desirability bias, scores on the PDCAS were significantly positively correlated with performance in the competition condition (but not the self condition) for female participants who believed they were competing against female confederates (*r* = 0.49, *p* = 0.009). This suggests that the more these participants viewed competition as a way to improve their skills, the better they performed in a competitive environment. However, given the small sample of female participants who competed against female confederates (*n* = 30), this finding may be very speculative. Furthermore, although one would then expect the PDCAS to be correlated with the number of shapes recalled from the competition condition, this finding was not significant. However, competitive performance scores (score during self condition subtracted from the score during the competition condition) did not predict competitive recall scores for females who believed they were competing against other females, suggesting that, although competition may increase performance for individuals who prefer competition as a means of improving performance, competitive performance does not very often translate to an increase in immediate long-term memory.

Overall, our results suggest that competition hindered working memory performance and immediate long-term memory for most groups in our task. The finding that competition may hinder memory is surprising; one explanation for this finding could be that the presence of a competitor could invoke high anxiety among participants, and high levels of anxiety have been shown to decrease working (Darke, 1988; Ashcraft and Kirk, 2001; Miller and Bichsel, 2004) and long-term memory (Rosenfeld, 1978; Cassady, 2004; Miller and Bichsel, 2004). Specifically, research has found that adolescents raised in high normative goal environments report the highest rates of competitive anxiety (White, 1998), which may lead to decrements in performance.

**FIGURE 6:**
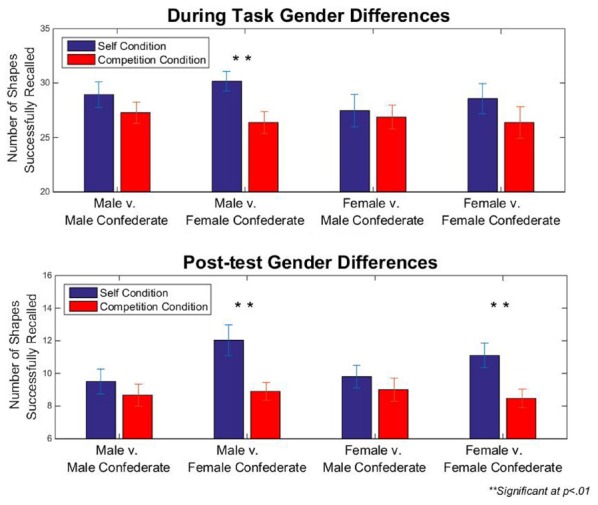
**Gender differences in Experiment 2.** Male and female participants performed worse in and recalled fewer shapes at post-test when they believed they were competing against female competitors. There were no significant differences for participants who believed they were competing again male competitors. Error bars reflect standard errors of the means.

Perhaps even more unanticipated is that the finding that the presence of a female competitor, but not a male, was most likely to hinder performance on our memory task. An alternative explanation for this finding would be that participants exerted less effort on the task because of the presence of a female competitor. However, because there was no significant difference involving gender, competition condition, and symmetry errors, these results suggest that the presence of a female competitor is more likely to be hindering processes involved in working memory—and subsequently, the processes necessary for encoding, as evident by the results of our recall task. Furthermore, we found significant differences between conditions for participants who believed they were competing against female confederates, but there was no significant interaction of gender by confederate gender. This may suggest that all participants may have reduced performance in the competition condition in a similar fashion (see Figure 6), and therefore not produced an interaction of gender by confederate gender.

Moreover, disparities in subjective reward could affect the memory processes required for learning, such as attention: succeeding in a competitive learning environment could feel subjectively more rewarding than succeeding in an individualist learning environment, and therefore distract participants’ attention, thereby disrupting working and long-term memory.

## General Discussion

### Competition, Attention, and Memory

Our results support the notion that a competitive environment can affect memory and effort. In Experiment 1, we examined the effect of competition on attention and effort; we found that the presence of a competitor increased attention on a physical effort task. However, we did not find that competition increased sustained effort on our task—just as competition did not affect the effort portion of Experiment 2 (symmetry matrices). This result could have occurred for a number of reasons: first, since RTs tend to be viewed as an implicit marker of motivation (Glaser and Knowles, 2008), perhaps competition affects effort on an implicit, rather than explicit, level, especially since our survey results suggest that participants tend to view overt competitive behavior as a negative trait. Second, perhaps competition is only salient enough to increase immediate attention in a laboratory setting, and not sustained physical effort on a task over time. More likely, however, competition may only affect performance on a physical effort task in an environment where competitors compete side-by-side, which did not occur in our task. Furthermore, Kilduff (2014) has found that competition tends to increase physical effort on a gross physical effort task (i.e., running a race). Nonetheless, the finding that competition may increase attention has crucial real-world applications for education and the workplace.

In Experiment 2, we examined the effects of the presence of a competitor on memory. Participants in our sample performed best on our working memory task in a non-competitive environment, and also learned more in a non-competitive environment, as demonstrated by their performance on a later recall test. These results could have occurred for a number of reasons. First, competition could be viewed as an anxiety-provoking threat for most participants: previous research has suggested that high levels of anxiety could have a negative effect on both working memory ability (Darke, 1988; Ashcraft and Kirk, 2001; Miller and Bichsel, 2004; Owens et al., 2012) and on learning (Rosenfeld, 1978; Cassady, 2004; Miller and Bichsel, 2004; Einsel and Turk, 2011). We would expect that, if participants viewed their competitor as a threat, this would indeed hinder performance, as was seen in our results. These findings were even stronger in our results regarding recall, suggesting that for most individuals, competition actually hinders memory. Furthermore, our sample consisted of students already at the undergraduate level of education, who may already be acclimated to cooperating with other students in academic settings (as opposed to competing). Since our sample consisted of U.S. undergraduate students—as opposed to students from a country such as Japan, in which competitive learning environments are common (Heine et al., 2001)—perhaps our participants were not adjusted to learning in a competitive environment. Competitive learning environments may have led to improvements in countries which have taught this way from an early age, suggesting that a competitive learning environment may be too novel for someone already at a higher level of education (Sanders, 1987; Smith, 1992).

Although competition improved initial RT in Experiment 1, the presence of a competitor hindered both working memory and immediate long-term memory in Experiment 2. Since attention is likely to increase both working memory (Awh et al., 2006; Berryhill et al., 2011) and learning (Nissen and Bullemer, 1987; Cohen et al., 1990; Gottlieb, 2012), why did this finding occur? It is possible that the difficulty of the task was responsible for this paradox: Experiment 1 featured a simple, button press task that required minimal effort. However, the multi-faceted task from Experiment 2 required more effort to succeed, and since greater emotional arousal may hinder performance and motivation on a very difficult task (Yerkes and Dodson, 1908; Watters et al., 1997; Diamond et al., 2007), it may be that the presence of a competitor was anxiety-provoking enough to hinder working memory performance and immediate long-term memory. In fact, previous research has found that RT tends to be faster after an increase in arousal, whereas executive tasks such as those necessary for successful working memory tend to benefit from a decrease in arousal (Luft et al., 2009). Furthermore, since competitive performance scores significantly predicted competitive recall scores, it may be that anxiety affected memory at the encoding phase—as opposed to affecting retention or retrieval.

An alternative explanation lies in the reward literature, as previous research has found that receiving rewards for a task can sometimes hinder performance, learning, and memory (Spence, 1970; McGraw and McCullers, 1974; Mobbs et al., 2009; Chib et al., 2012). Perhaps succeeding in a competitive learning environment was subjectively more rewarding than succeeding in an individualist setting, despite objective rewards remaining the same across conditions. If succeeding in a competitive learning environment is subjectively more rewarding than succeeding in an individualist setting, competition may be more likely to distract participants—similarly to “choking under pressure” (Baumeister, 1984; Beilock and Carr, 2001, 2005; Ramirez et al., 2013). This explanation may be why competition negatively affecting working memory and immediate long-term memory on our task. There also may individual differences in preferences for competitive learning environments. In future research, it would be valuable to discern participants’ preference for the competition condition, as this information may provide insight as to the possible distractibility of competition and memory.

### Individual and Gender Differences

In Experiment 1, we found that the PDCAS predicted how competitive an individual was at an effort bar task. In Experiment 2, the PDCAS predicted how competitive an individual was in a memory task, although this finding did not remain significant after correcting for multiple comparisons. Competitiveness in a learning setting is likely to be contingent on more factors than can be grasped from one survey measure. Furthermore, we found that men scored significantly higher on the PDCAS, suggesting that men may value competition as a means for improving personal development more than women. Men also exhibited a more competitive performance in our physical effort task in Experiment 1, in line with recent research that suggests men tend to both prefer and perform better in competitive physical environments more so than women (Gneezy et al., 2009; Niederle and Vesterlund, 2011). However, men did not outperform women in our repeated memory task in Experiment 2. Competition may affect performance on memory tasks differently than competition traditionally affects effort and attention. Furthermore, since previous studies [such as Gneezy et al. (2009)] have typically utilized effort tasks to compare preference for competitive environments, future research studies may want to further examine gender differences in preference for competition in memory tasks specifically, since these are typically utilized in educational settings.

We also found high rates of social desirability in our sample, which was negatively correlated with the HAS—but not the PDCAS—suggesting that the PDCAS may be a superior survey measure when tapping an individual’s true trait competitive habits and preferences. Furthermore, because the HAS contains blatant questions regarding competition, its negative correlation with social desirability may suggest that competition may be viewed as a negative personality trait by most individuals.

In Experiment 2, we found significant differences in performance on a memory task when a participant believed they were competing against a female participant. However, this result was not the case in Experiment 1 in a physical effort task. Although some research has found that females tend to excel at tasks involving episodic memory (Herlitz et al., 1997; Davis, 1999) and object identification memory tasks (Voyer et al., 2007), which were strong skills necessary to succeed at the type of task used in Experiment 2, whether this gender advantage was known by our participants remains unknown. Research suggests that increased attention drawn to one’s own performance can result in performance decrements or “choking under pressure” (Baumeister, 1984; Beilock and Carr, 2001, 2005; Ramirez et al., 2013), so the presence of a female competitor may increase pressure in a learning environment if participants have had previous experience with an object identification memory tasks and a female rivals, such as in a classroom learning setting. Yet, it is unclear whether the performance differences we found among participants who believed they were competing against female competitors were due to increased pressure due to the presence of a female competitor, or the opposite view: that females did not appear to be strong opponents in a learning setting, so they did not cause their competitors to devote more attentional resources to the task. However, although we found significant differences between conditions for participants who believed they were competing against female confederates, there was no significant interaction of gender by confederate gender, suggesting that all participants may have reduced performance in the competition condition.

### Limitations

It may be difficult to generalize our experiment to competition and memory in a real-world sense. Our task in Experiment 1 examined how social motivation’s effect on a simple physical effort task, but competition may affect gross physical effort (e.g., running, team sports, etc.) on a more complex level. Additionally, our task from Experiment 2 was a specific, short memory task that did not offer any realistic long-term gains. Future research should include a longer period before administering a recall task, as a longer delay before recall would more realistically illustrate how learning occurs in a classroom setting. Furthermore, although individual preferences in competition were obtained, individual differences in intrinsic vs. extrinsic reward preference were not accounted for, and an additional sum of a few dollars may not have been enough motivation for some individuals to increase performance. Future research should examine how competition may influence long-term memory in a true educational setting.

Because our study examined the effect of competition on memory in two tasks that also featured gains and losses, our findings may have been driven by the effect of gains and losses on attention and performance, moderated by the saliency of a competitor. Since previous research has suggested that losses can increase both attention and performance (Yechiam and Hochman, 2013), future research studies should attempt to distinguish whether or not competition merely moderates this affect, especially since most competitive learning environments incorporate some type of gains and losses, such as in educational settings.

## Conclusion

In sum, our research suggests that social motivation—specifically, competition—can have strong effects on attention and memory, although significant individual and gender differences exist. Competition in a physical effort setting may increase attention, while the presence of a competitor may have detrimental effects on memory and performance. These findings present strong implications for education, the workplace, and other real-world settings involving social interaction.

### Conflict of Interest Statement

The authors declare that the research was conducted in the absence of any commercial or financial relationships that could be construed as a potential conflict of interest.
